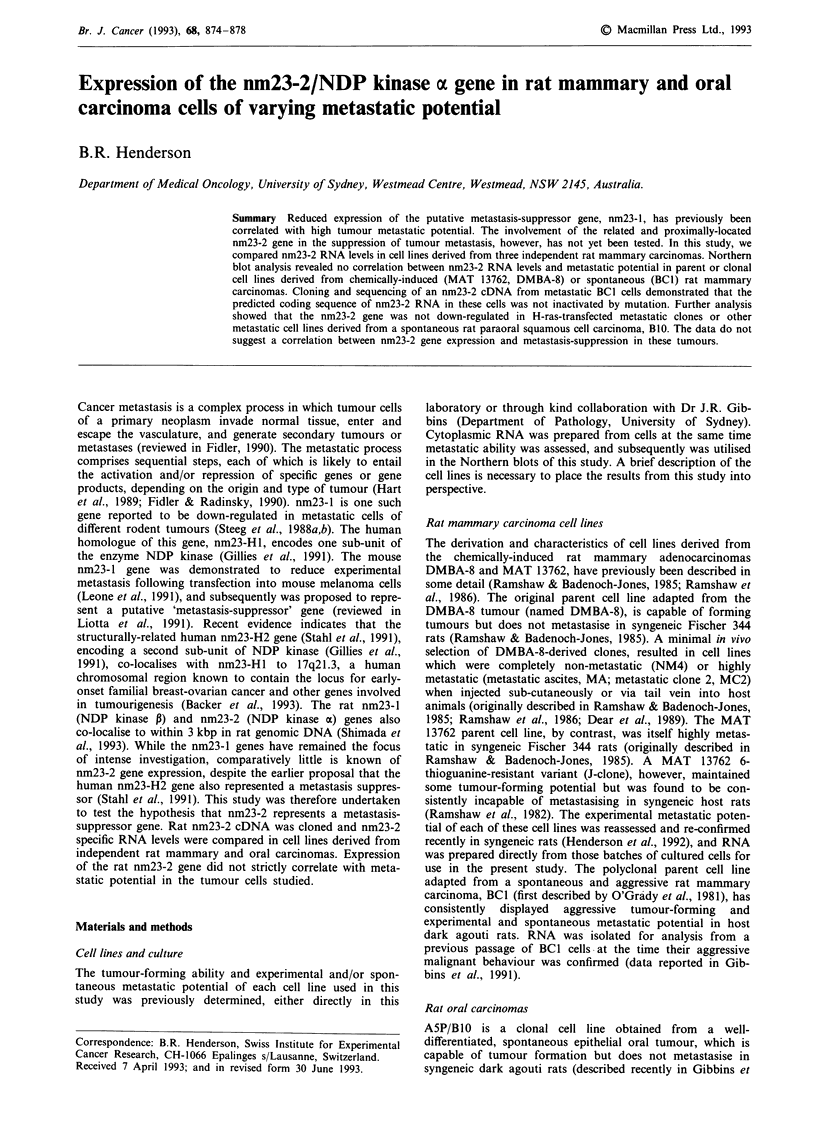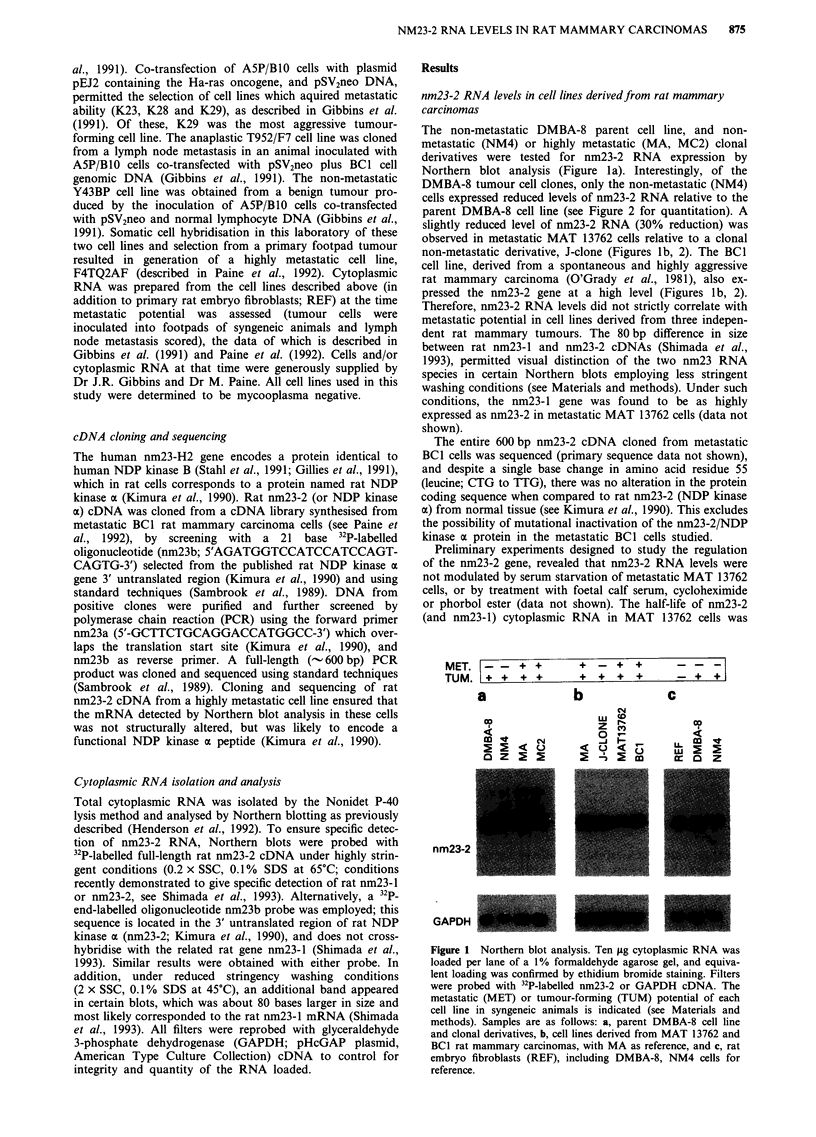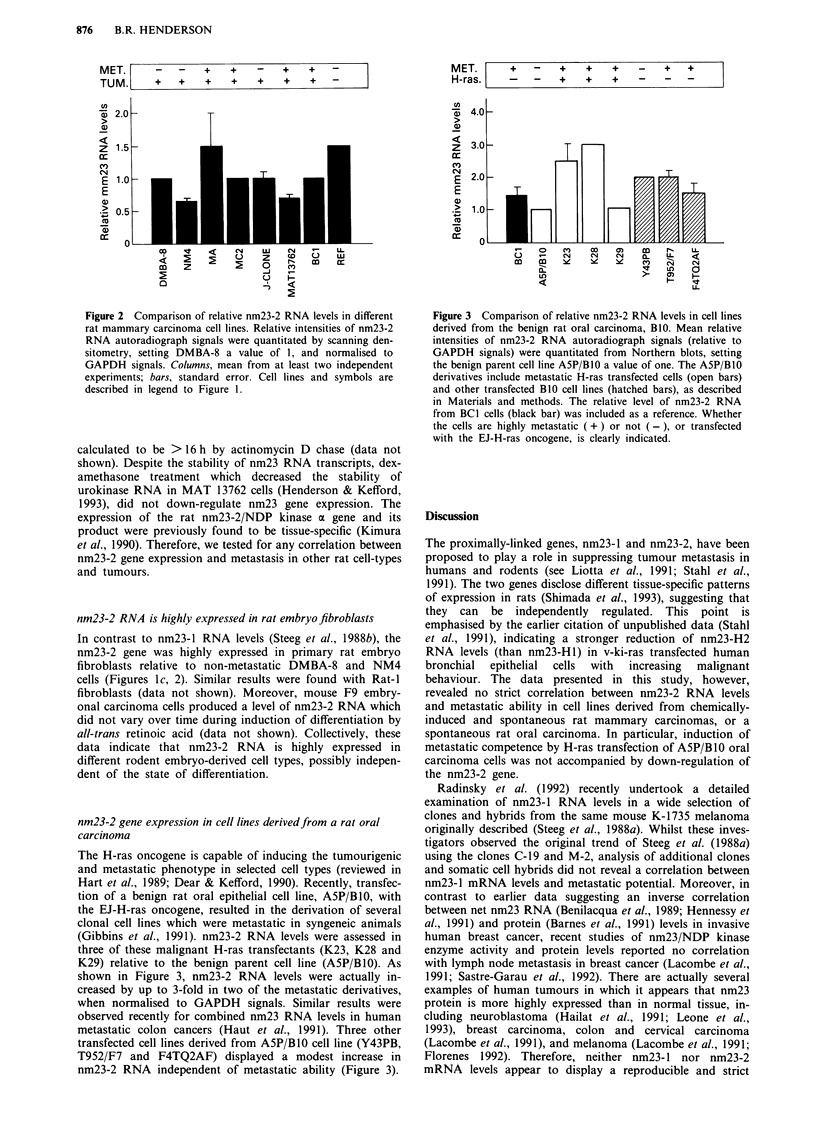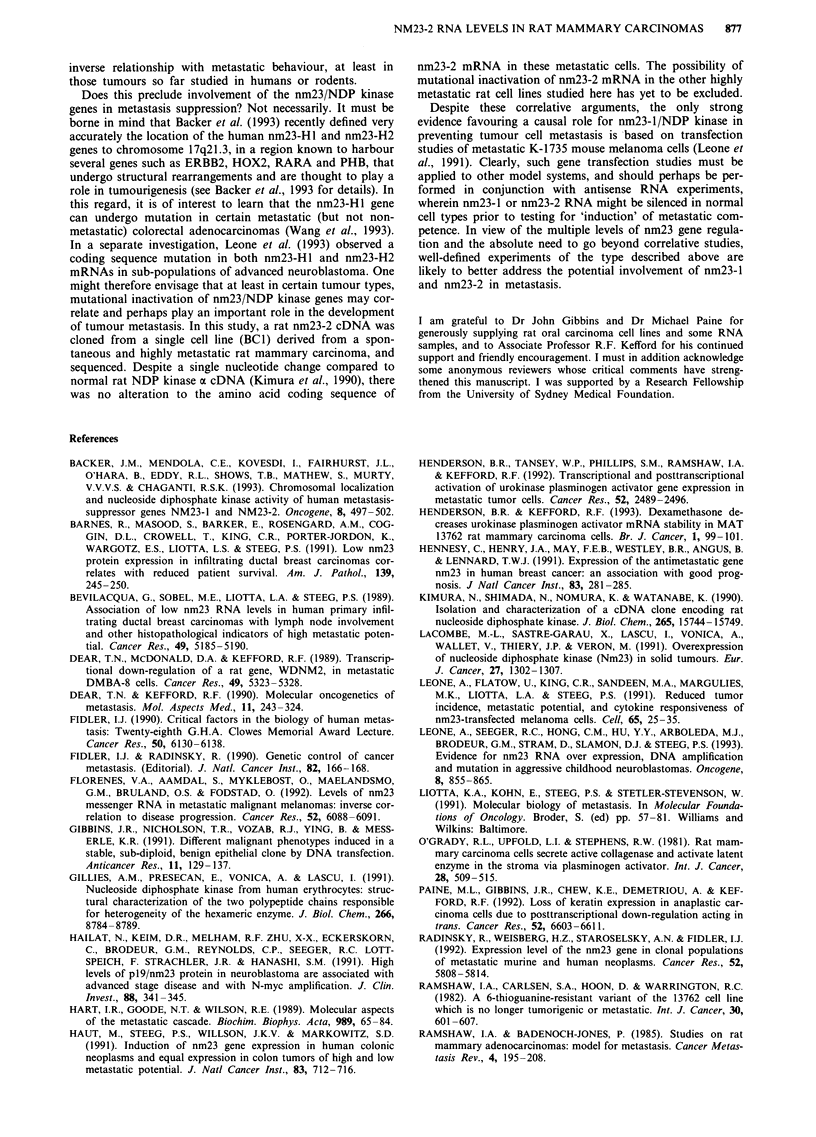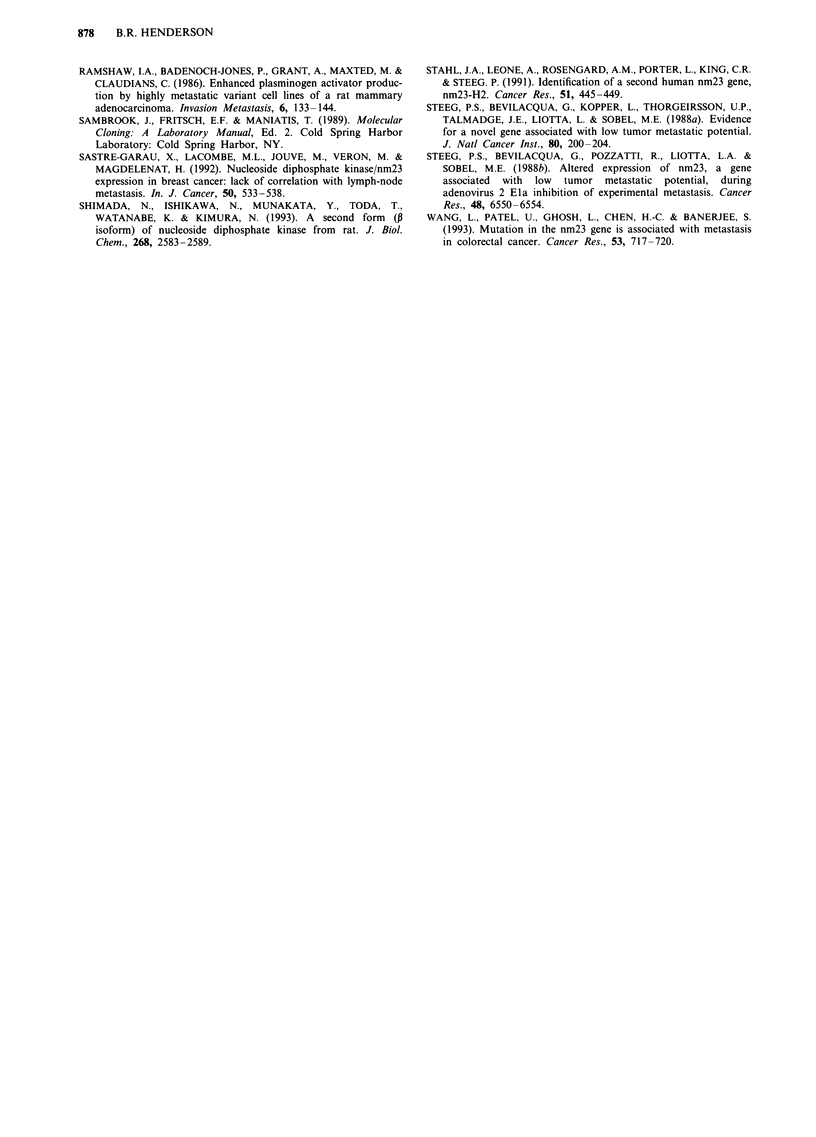# Expression of the nm23-2/NDP kinase alpha gene in rat mammary and oral carcinoma cells of varying metastatic potential.

**DOI:** 10.1038/bjc.1993.448

**Published:** 1993-11

**Authors:** B. R. Henderson

**Affiliations:** Department of Medical Oncology, University of Sydney, Westmead Centre, NSW, Australia.

## Abstract

**Images:**


*Br. J. Cancer* (1993), **68**, 874–878
© Macmillan Press Ltd., 1993

### Expression of the nm23-2/NDP kinase α gene in rat mammary and oral carcinoma cells of varying metastatic potential

B.R. Henderson

*Department of Medical Oncology, University of Sydney, Westmead Centre, Westmead, NSW 2145, Australia.*

**Summary** Reduced expression of the putative metastasis-suppressor gene, nm23-1, has previously been correlated with high tumour metastatic potential. The involvement of the related and proximally-located nm23-2 gene in the suppression of tumour metastasis, however, has not yet been tested. In this study, we compared nm23-2 RNA levels in cell lines derived from three independent rat mammary carcinomas. Northern blot analysis revealed no correlation between nm23-2 RNA levels and metastatic potential in parent or clonal cell lines derived from chemically-induced (MAT 13762, DMBA-8) or spontaneous (BC1) rat mammary carcinomas. Cloning and sequencing of an nm23-2 cDNA from metastatic BC1 cells demonstrated that the predicted coding sequence of nm23-2 RNA in these cells was not inactivated by mutation. Further analysis showed that the nm23-2 gene was not down-regulated in H-ras-transfected metastatic clones or other metastatic cell lines derived from a spontaneous rat paraoral squamous cell carcinoma, B10. The data do not suggest a correlation between nm23-2 gene expression and metastasis-suppression in these tumours.

Cancer metastasis is a complex process in which tumour cells of a primary neoplasm invade normal tissue, enter and escape the vasculature, and generate secondary tumours or metastases (reviewed in Fidler, 1990). The metastatic process comprises sequential steps, each of which is likely to entail the activation and/or repression of specific genes or gene products, depending on the origin and type of tumour (Hart *et al.*, 1989; Fidler & Radinsky, 1990). nm23-1 is one such gene reported to be down-regulated in metastatic cells of different rodent tumours (Steeg *et al.*, 1988*a,b*). The human homologue of this gene, nm23-H1, encodes one sub-unit of the enzyme NDP kinase (Gillies *et al.*, 1991). The mouse nm23-1 gene was demonstrated to reduce experimental metastasis following transfection into mouse melanoma cells (Leone *et al.*, 1991), and subsequently was proposed to represent a putative 'metastasis-suppressor' gene (reviewed in Liotta *et al.*, 1991). Recent evidence indicates that the structurally-related human nm23-H2 gene (Stahl *et al.*, 1991), encoding a second sub-unit of NDP kinase (Gillies *et al.*, 1991), co-localises with nm23-H1 to 17q21.3, a human chromosomal region known to contain the locus for early-onset familial breast-ovarian cancer and other genes involved in tumourigenesis (Backer *et al.*, 1993). The rat nm23-1 (NDP kinase β) and nm23-2 (NDP kinase α) genes also co-localise to within 3 kbp in rat genomic DNA (Shimada *et al.*, 1993). While the nm23-1 genes have remained the focus of intense investigation, comparatively little is known of nm23-2 gene expression, despite the earlier proposal that the human nm23-H2 gene also represented a metastasis suppressor (Stahl *et al.*, 1991). This study was therefore undertaken to test the hypothesis that nm23-2 represents a metastasis-suppressor gene. Rat nm23-2 cDNA was cloned and nm23-2 specific RNA levels were compared in cell lines derived from independent rat mammary and oral carcinomas. Expression of the rat nm23-2 gene did not strictly correlate with metastatic potential in the tumour cells studied.

### Materials and methods

### *Cell lines and culture*

The tumour-forming ability and experimental and/or spontaneous metastatic potential of each cell line used in this study was previously determined, either directly in this laboratory or through kind collaboration with Dr J.R. Gibbins (Department of Pathology, University of Sydney). Cytoplasmic RNA was prepared from cells at the same time metastatic ability was assessed, and subsequently was utilised in the Northern blots of this study. A brief description of the cell lines is necessary to place the results from this study into perspective.

### *Rat mammary carcinoma cell lines*

The derivation and characteristics of cell lines derived from the chemically-induced rat mammary adenocarcinomas DMBA-8 and MAT 13762, have previously been described in some detail (Ramshaw & Badenoch-Jones, 1985; Ramshaw *et al.*, 1986). The original parent cell line adapted from the DMBA-8 tumour (named DMBA-8), is capable of forming tumours but does not metastasise in syngeneic Fischer 344 rats (Ramshaw & Badenoch-Jones, 1985). A minimal *in vivo* selection of DMBA-8-derived clones, resulted in cell lines which were completely non-metastatic (NM4) or highly metastatic (metastatic ascites, MA; metastatic clone 2, MC2) when injected sub-cutaneously or via tail vein into host animals (originally described in Ramshaw & Badenoch-Jones, 1985; Ramshaw *et al.*, 1986; Dear *et al.*, 1989). The MAT 13762 parent cell line, by contrast, was itself highly metastatic in syngeneic Fischer 344 rats (originally described in Ramshaw & Badenoch-Jones, 1985). A MAT 13762 6-thioguanine-resistant variant (J-clone), however, maintained some tumour-forming potential but was found to be consistently incapable of metastasising in syngeneic host rats (Ramshaw *et al.*, 1982). The experimental metastatic potential of each of these cell lines was reassessed and re-confirmed recently in syngeneic rats (Henderson *et al.*, 1992), and RNA was prepared directly from those batches of cultured cells for use in the present study. The polyclonal parent cell line adapted from a spontaneous and aggressive rat mammary carcinoma, BC1 (first described by O'Grády *et al.*, 1981), has consistently displayed aggressive tumour-forming and experimental and spontaneous metastatic potential in host dark agouti rats. RNA was isolated for analysis from a previous passage of BC1 cells at the time their aggressive malignant behaviour was confirmed (data reported in Gibbins *et al.*, 1991).

### *Rat oral carcinomas*

A5P/B10 is a clonal cell line obtained from a well-differentiated, spontaneous epithelial oral tumour, which is capable of tumour formation but does not metastasise in syngeneic dark agouti rats (described recently in Gibbins *et*

Correspondence: B.R. Henderson, Swiss Institute for Experimental Cancer Research, CH-1066 Epalinges s/Lausanne, Switzerland.
Received 7 April 1993; and in revised form 30 June 1993.

*al.*, 1991). Co-transfection of A5P/B10 cells with plasmid pEJ2 containing the Ha-ras oncogene, and $pSV_2neo$ DNA, permitted the selection of cell lines which aquired metastatic ability (K23, K28 and K29), as described in Gibbins *et al.* (1991). Of these, K29 was the most aggressive tumour-forming cell line. The anaplastic T952/F7 cell line was cloned from a lymph node metastasis in an animal inoculated with A5P/B10 cells co-transfected with $pSV_2neo$ plus BC1 cell genomic DNA (Gibbins *et al.*, 1991). The non-metastatic Y43BP cell line was obtained from a benign tumour produced by the inoculation of A5P/B10 cells co-transfected with $pSV_2neo$ and normal lymphocyte DNA (Gibbins *et al.*, 1991). Somatic cell hybridisation in this laboratory of these two cell lines and selection from a primary footpad tumour resulted in generation of a highly metastatic cell line, F4TQ2AF (described in Paine *et al.*, 1992). Cytoplasmic RNA was prepared from the cell lines described above (in addition to primary rat embryo fibroblasts; REF) at the time metastatic potential was assessed (tumour cells were inoculated into footpads of syngeneic animals and lymph node metastasis scored), the data of which is described in Gibbins *et al.* (1991) and Paine *et al.* (1992). Cells and/or cytoplasmic RNA at that time were generously supplied by Dr J.R. Gibbins and Dr M. Paine. All cell lines used in this study were determined to be mycooplasma negative.

### *cDNA cloning and sequencing*

The human nm23-H2 gene encodes a protein identical to human NDP kinase B (Stahl *et al.*, 1991; Gillies *et al.*, 1991), which in rat cells corresponds to a protein named rat NDP kinase α (Kimura *et al.*, 1990). Rat nm23-2 (or NDP kinase α) cDNA was cloned from a cDNA library synthesised from metastatic BC1 rat mammary carcinoma cells (see Paine *et al.*, 1992), by screening with a 21 base $^{32}P$-labelled oligonucleotide (nm23b; 5′AGATGGTCCATCCATCCAGT-CAGTG-3′) selected from the published rat NDP kinase α gene 3′ untranslated region (Kimura *et al.*, 1990) and using standard techniques (Sambrook *et al.*, 1989). DNA from positive clones were purified and further screened by polymerase chain reaction (PCR) using the forward primer nm23a (5′-GCTTCTGCAGGACCATGGCC-3′) which overlaps the translation start site (Kimura *et al.*, 1990), and nm23b as reverse primer. A full-length (~600 bp) PCR product was cloned and sequenced using standard techniques (Sambrook *et al.*, 1989). Cloning and sequencing of rat nm23-2 cDNA from a highly metastatic cell line ensured that the mRNA detected by Northern blot analysis in these cells was not structurally altered, but was likely to encode a functional NDP kinase α peptide (Kimura *et al.*, 1990).

### *Cytoplasmic RNA isolation and analysis*

Total cytoplasmic RNA was isolated by the Nonidet P-40 lysis method and analysed by Northern blotting as previously described (Henderson *et al.*, 1992). To ensure specific detection of nm23-2 RNA, Northern blots were probed with $^{32}P$-labelled full-length rat nm23-2 cDNA under highly stringent conditions (0.2 × SSC, 0.1% SDS at 65°C; conditions recently demonstrated to give specific detection of rat nm23-1 or nm23-2, see Shimada *et al.*, 1993). Alternatively, a $^{32}P$-end-labelled oligonucleotide nm23b probe was employed; this sequence is located in the 3′ untranslated region of rat NDP kinase α (nm23-2; Kimura *et al.*, 1990), and does not cross-hybridise with the related rat gene nm23-1 (Shimada *et al.*, 1993). Similar results were obtained with either probe. In addition, under reduced stringency washing conditions (2 × SSC, 0.1% SDS at 45°C), an additional band appeared in certain blots, which was about 80 bases larger in size and most likely corresponded to the rat nm23-1 mRNA (Shimada *et al.*, 1993). All filters were reprobed with glyceraldehyde 3-phosphate dehydrogenase (GAPDH; pHcGAP plasmid, American Type Culture Collection) cDNA to control for integrity and quantity of the RNA loaded.

### Results

### *nm23-2 RNA levels in cell lines derived from rat mammary carcinomas*

The non-metastatic DMBA-8 parent cell line, and non-metastatic (NM4) or highly metastatic (MA, MC2) clonal derivatives were tested for nm23-2 RNA expression by Northern blot analysis (Figure 1a). Interestingly, of the DMBA-8 tumour cell clones, only the non-metastatic (NM4) cells expressed reduced levels of nm23-2 RNA relative to the parent DMBA-8 cell line (see Figure 2 for quantitation). A slightly reduced level of nm23-2 RNA (30% reduction) was observed in metastatic MAT 13762 cells relative to a clonal non-metastatic derivative, J-clone (Figures 1b, 2). The BC1 cell line, derived from a spontaneous and highly aggressive rat mammary carcinoma (O'Grady *et al.*, 1981), also expressed the nm23-2 gene at a high level (Figures 1b, 2). Therefore, nm23-2 RNA levels did not strictly correlate with metastatic potential in cell lines derived from three independent rat mammary tumours. The 80 bp difference in size between rat nm23-1 and nm23-2 cDNAs (Shimada *et al.*, 1993), permitted visual distinction of the two nm23 RNA species in certain Northern blots employing less stringent washing conditions (see Materials and methods). Under such conditions, the nm23-1 gene was found to be as highly expressed as nm23-2 in metastatic MAT 13762 cells (data not shown).

The entire 600 bp nm23-2 cDNA cloned from metastatic BC1 cells was sequenced (primary sequence data not shown), and despite a single base change in amino acid residue 55 (leucine; CTG to TTG), there was no alteration in the protein coding sequence when compared to rat nm23-2 (NDP kinase α) from normal tissue (see Kimura *et al.*, 1990). This excludes the possibility of mutational inactivation of the nm23-2/NDP kinase α protein in the metastatic BC1 cells studied.

Preliminary experiments designed to study the regulation of the nm23-2 gene, revealed that nm23-2 RNA levels were not modulated by serum starvation of metastatic MAT 13762 cells, or by treatment with foetal calf serum, cycloheximide or phorbol ester (data not shown). The half-life of nm23-2 (and nm23-1) cytoplasmic RNA in MAT 13762 cells was

**Figure 1** Northern blot analysis. Ten μg cytoplasmic RNA was loaded per lane of a 1% formaldehyde agarose gel, and equivalent loading was confirmed by ethidium bromide staining. Filters were probed with $^{32}P$-labelled nm23-2 or GAPDH cDNA. The metastatic (MET) or tumour-forming (TUM) potential of each cell line in syngeneic animals is indicated (see Materials and methods). Samples are as follows: **a**, parent DMBA-8 cell line and clonal derivatives, **b**, cell lines derived from MAT 13762 and BC1 rat mammary carcinomas, with MA as reference, and **c**, rat embryo fibroblasts (REF), including DMBA-8, NM4 cells for reference.

**Figure 2** Comparison of relative nm23-2 RNA levels in different rat mammary carcinoma cell lines. Relative intensities of nm23-2 RNA autoradiograph signals were quantitated by scanning densitometry, setting DMBA-8 a value of 1, and normalised to GAPDH signals. *Columns*, mean from at least two independent experiments; *bars*, standard error. Cell lines and symbols are described in legend to Figure 1.

**Figure 3** Comparison of relative nm23-2 RNA levels in cell lines derived from the benign rat oral carcinoma, B10. Mean relative intensities of nm23-2 RNA autoradiograph signals (relative to GAPDH signals) were quantitated from Northern blots, setting the benign parent cell line A5P/B10 a value of one. The A5P/B10 derivatives include metastatic H-ras transfected cells (open bars) and other transfected B10 cell lines (hatched bars), as described in Materials and methods. The relative level of nm23-2 RNA from BC1 cells (black bar) was included as a reference. Whether the cells are highly metastatic ( + ) or not ( – ), or transfected with the EJ-H-ras oncogene, is clearly indicated.

calculated to be > 16 h by actinomycin D chase (data not shown). Despite the stability of nm23 RNA transcripts, dexamethasone treatment which decreased the stability of urokinase RNA in MAT 13762 cells (Henderson & Kefford, 1993), did not down-regulate nm23 gene expression. The expression of the rat nm23-2/NDP kinase α gene and its product were previously found to be tissue-specific (Kimura *et al.*, 1990). Therefore, we tested for any correlation between nm23-2 gene expression and metastasis in other rat cell-types and tumours.

### *nm23-2 RNA is highly expressed in rat embryo fibroblasts*

In contrast to nm23-1 RNA levels (Steeg *et al.*, 1988*b*), the nm23-2 gene was highly expressed in primary rat embryo fibroblasts relative to non-metastatic DMBA-8 and NM4 cells (Figures 1*c*, 2). Similar results were found with Rat-1 fibroblasts (data not shown). Moreover, mouse F9 embryonal carcinoma cells produced a level of nm23-2 RNA which did not vary over time during induction of differentiation by *all-trans* retinoic acid (data not shown). Collectively, these data indicate that nm23-2 RNA is highly expressed in different rodent embryo-derived cell types, possibly independent of the state of differentiation.

### *nm23-2 gene expression in cell lines derived from a rat oral carcinoma*

The H-ras oncogene is capable of inducing the tumourigenic and metastatic phenotype in selected cell types (reviewed in Hart *et al.*, 1989; Dear & Kefford, 1990). Recently, transfection of a benign rat oral epithelial cell line, A5P/B10, with the EJ-H-ras oncogene, resulted in the derivation of several clonal cell lines which were metastatic in syngeneic animals (Gibbins *et al.*, 1991). nm23-2 RNA levels were assessed in three of these malignant H-ras transfectants (K23, K28 and K29) relative to the benign parent cell line (A5P/B10). As shown in Figure 3, nm23-2 RNA levels were actually increased by up to 3-fold in two of the metastatic derivatives, when normalised to GAPDH signals. Similar results were observed recently for combined nm23 RNA levels in human metastatic colon cancers (Haut *et al.*, 1991). Three other transfected cell lines derived from A5P/B10 cell line (Y43PB, T952/F7 and F4TQ2AF) displayed a modest increase in nm23-2 RNA independent of metastatic ability (Figure 3).

### Discussion

The proximally-linked genes, nm23-1 and nm23-2, have been proposed to play a role in suppressing tumour metastasis in humans and rodents (see Liotta *et al.*, 1991; Stahl *et al.*, 1991). The two genes disclose different tissue-specific patterns of expression in rats (Shimada *et al.*, 1993), suggesting that they can be independently regulated. This point is emphasised by the earlier citation of unpublished data (Stahl *et al.*, 1991), indicating a stronger reduction of nm23-H2 RNA levels (than nm23-H1) in v-ki-ras transfected human bronchial epithelial cells with increasing malignant behaviour. The data presented in this study, however, revealed no strict correlation between nm23-2 RNA levels and metastatic ability in cell lines derived from chemically-induced and spontaneous rat mammary carcinomas, or a spontaneous rat oral carcinoma. In particular, induction of metastatic competence by H-ras transfection of A5P/B10 oral carcinoma cells was not accompanied by down-regulation of the nm23-2 gene.

Radinsky *et al.* (1992) recently undertook a detailed examination of nm23-1 RNA levels in a wide selection of clones and hybrids from the same mouse K-1735 melanoma originally described (Steeg *et al.*, 1988*a*). Whilst these investigators observed the original trend of Steeg *et al.* (1988*a*) using the clones C-19 and M-2, analysis of additional clones and somatic cell hybrids did not reveal a correlation between nm23-1 mRNA levels and metastatic potential. Moreover, in contrast to earlier data suggesting an inverse correlation between net nm23 RNA (Benilacqua *et al.*, 1989; Hennessy *et al.*, 1991) and protein (Barnes *et al.*, 1991) levels in invasive human breast cancer, recent studies of nm23/NDP kinase enzyme activity and protein levels reported no correlation with lymph node metastasis in breast cancer (Lacombe *et al.*, 1991; Sastre-Garau *et al.*, 1992). There are actually several examples of human tumours in which it appears that nm23 protein is more highly expressed than in normal tissue, including neuroblastoma (Hailat *et al.*, 1991; Leone *et al.*, 1993), breast carcinoma, colon and cervical carcinoma (Lacombe *et al.*, 1991), and melanoma (Lacombe *et al.*, 1991; Florenes 1992). Therefore, neither nm23-1 nor nm23-2 mRNA levels appear to display a reproducible and strict inverse relationship with metastatic behaviour, at least in those tumours so far studied in humans or rodents.

Does this preclude involvement of the nm23/NDP kinase genes in metastasis suppression? Not necessarily. It must be borne in mind that Backer *et al.* (1993) recently defined very accurately the location of the human nm23-H1 and nm23-H2 genes to chromosome 17q21.3, in a region known to harbour several genes such as ERBB2, HOX2, RARA and PHB, that undergo structural rearrangements and are thought to play a role in tumourigenesis (see Backer *et al.*, 1993 for details). In this regard, it is of interest to learn that the nm23-H1 gene can undergo mutation in certain metastatic (but not non-metastatic) colorectal adenocarcinomas (Wang *et al.*, 1993). In a separate investigation, Leone *et al.* (1993) observed a coding sequence mutation in both nm23-H1 and nm23-H2 mRNAs in sub-populations of advanced neuroblastoma. One might therefore envisage that at least in certain tumour types, mutational inactivation of nm23/NDP kinase genes may correlate and perhaps play an important role in the development of tumour metastasis. In this study, a rat nm23-2 cDNA was cloned from a single cell line (BC1) derived from a spontaneous and highly metastatic rat mammary carcinoma, and sequenced. Despite a single nucleotide change compared to normal rat NDP kinase α cDNA (Kimura *et al.*, 1990), there was no alteration to the amino acid coding sequence of nm23-2 mRNA in these metastatic cells. The possibility of mutational inactivation of nm23-2 mRNA in the other highly metastatic rat cell lines studied here has yet to be excluded.

Despite these correlative arguments, the only strong evidence favouring a causal role for nm23-1/NDP kinase in preventing tumour cell metastasis is based on transfection studies of metastatic K-1735 mouse melanoma cells (Leone *et al.*, 1991). Clearly, such gene transfection studies must be applied to other model systems, and should perhaps be performed in conjunction with antisense RNA experiments, wherein nm23-1 or nm23-2 RNA might be silenced in normal cell types prior to testing for 'induction' of metastatic competence. In view of the multiple levels of nm23 gene regulation and the absolute need to go beyond correlative studies, well-defined experiments of the type described above are likely to better address the potential involvement of nm23-1 and nm23-2 in metastasis.

I am grateful to Dr John Gibbins and Dr Michael Paine for generously supplying rat oral carcinoma cell lines and some RNA samples, and to Associate Professor R.F. Kefford for his continued support and friendly encouragement. I must in addition acknowledge some anonymous reviewers whose critical comments have strengthened this manuscript. I was supported by a Research Fellowship from the University of Sydney Medical Foundation.

### References

BACKER, J.M., MENDOLA, C.E., KOVESDI, I., FAIRHURST, J.L., O'HARA, B., EDDY, R.L., SHOWS, T.B., MATHEW, S., MURTY, V.V.V.S. & CHAGANTI, R.S.K. (1993). Chromosomal localization and nucleoside diphosphate kinase activity of human metastasis-suppressor genes NM23-1 and NM23-2. *Oncogene*, **8**, 497–502.

BARNES, R., MASOOD, S., BARKER, E., ROSENGARD, A.M., COGGIN, D.L., CROWELL, T., KING, C.R., PORTER-JORDON, K., WARGOTZ, E.S., LIOTTA, L.S. & STEEG, P.S. (1991). Low nm23 protein expression in infiltrating ductal breast carcinomas correlates with reduced patient survival. *Am. J. Pathol.*, **139**, 245–250.

BEVILACQUA, G., SOBEL, M.E., LIOTTA, L.A. & STEEG, P.S. (1989). Association of low nm23 RNA levels in human primary infiltrating ductal breast carcinomas with lymph node involvement and other histopathological indicators of high metastatic potential. *Cancer Res.*, **49**, 5185–5190.

DEAR, T.N., McDONALD, D.A. & KEFFORD, R.F. (1989). Transcriptional down-regulation of a rat gene, WDNM2, in metastatic DMBA-8 cells. *Cancer Res.*, **49**, 5323–5328.

DEAR, T.N. & KEFFORD, R.F. (1990). Molecular oncogenetics of metastasis. *Mol. Aspects Med.*, **11**, 243–324.

FIDLER, I.J. (1990). Critical factors in the biology of human metastasis: Twenty-eighth G.H.A. Clowes Memorial Award Lecture. *Cancer Res.*, **50**, 6130–6138.

FIDLER, I.J. & RADINSKY, R. (1990). Genetic control of cancer metastasis. (Editorial). *J. Natl. Cancer Inst.*, **82**, 166–168.

FLORENES, V.A., AAMDAL, S., MYKLEBOST, O., MAELANDSMO, G.M., BRULAND, O.S. & FODSTAD, O. (1992). Levels of nm23 messenger RNA in metastatic malignant melanomas: inverse correlation to disease progression. *Cancer Res.*, **52**, 6088–6091.

GIBBINS, J.R., NICHOLSON, T.R., VOZAB, R.J., YING, B. & MESSERLE, K.R. (1991). Different malignant phenotypes induced in a stable, sub-diploid, benign epithelial clone by DNA transfection. *Anticancer Res.*, **11**, 129–137.

GILLIES, A.M., PRESECAN, E., VONICA, A. & LASCU, I. (1991). Nucleoside diphosphate kinase from human erythrocytes: structural characterization of the two polypeptide chains responsible for heterogeneity of the hexameric enzyme. *J. Biol. Chem.*, **266**, 8784–8789.

HAILAT, N., KEIM, D.R., MELHAM, R.F. ZHU, X-X., ECKERSKORN, C., BRODEUR, G.M., REYNOLDS, C.P., SEEGER, R.C. LOTTSPEICH, F. STRACHLER, J.R. & HANASHI, S.M. (1991). High levels of p19/nm23 protein in neuroblastoma are associated with advanced stage disease and with N-myc amplification. *J. Clin. Invest.*, **88**, 341–345.

HART, I.R., GOODE, N.T. & WILSON, R.E. (1989). Molecular aspects of the metastatic cascade. *Biochim. Biophys. Acta*, **989**, 65–84.

HAUT, M., STEEG, P.S., WILLSON, J.K.V. & MARKOWITZ, S.D. (1991). Induction of nm23 gene expression in human colonic neoplasms and equal expression in colon tumors of high and low metastatic potential. *J. Natl Cancer Inst.*, **83**, 712–716.

HENDERSON, B.R., TANSEY, W.P., PHILLIPS, S.M., RAMSHAW, I.A. & KEFFORD, R.F. (1992). Transcriptional and posttranscriptional activation of urokinase plasminogen activator gene expression in metastatic tumor cells. *Cancer Res.*, **52**, 2489–2496.

HENDERSON, B.R. & KEFFORD, R.F. (1993). Dexamethasone decreases urokinase plasminogen activator mRNA stability in MAT 13762 rat mammary carcinoma cells. *Br. J. Cancer*, **1**, 99–101.

HENNESY, C., HENRY, J.A., MAY, F.E.B., WESTLEY, B.R., ANGUS, B. & LENNARD, T.W.J. (1991). Expression of the antimetastatic gene nm23 in human breast cancer: an association with good prognosis. *J Natl Cancer Inst.*, **83**, 281–285.

KIMURA, N., SHIMADA, N., NOMURA, K. & WATANABE, K. (1990). Isolation and characterization of a cDNA clone encoding rat nucleoside diphosphate kinase. *J. Biol. Chem.*, **265**, 15744–15749.

LACOMBE, M.-L., SASTRE-GARAU, X., LASCU, I., VONICA, A., WALLET, V., THIERY, J.P. & VERON, M. (1991). Overexpression of nucleoside diphosphate kinase (Nm23) in solid tumours. *Eur. J. Cancer*, **27**, 1302–1307.

LEONE, A., FLATOW, U., KING, C.R., SANDEEN, M.A., MARGULIES, M.K., LIOTTA, L.A. & STEEG, P.S. (1991). Reduced tumor incidence, metastatic potential, and cytokine responsiveness of nm23-transfected melanoma cells. *Cell*, **65**, 25–35.

LEONE, A., SEEGER, R.C., HONG, C.M., HU, Y.Y., ARBOLEDA, M.J., BRODEUR, G.M., STRAM, D., SLAMON, D.J. & STEEG, P.S. (1993). Evidence for nm23 RNA over expression, DNA amplification and mutation in aggressive childhood neuroblastomas. *Oncogene*, **8**, 855–865.

LIOTTA, K.A., KOHN, E., STEEG, P.S. & STETLER-STEVENSON, W. (1991). Molecular biology of metastasis. In *Molecular Foundations of Oncology*. Broder, S. (ed) pp. 57–81. Williams and Wilkins: Baltimore.

O'GRADY, R.L., UPFOLD, L.I. & STEPHENS, R.W. (1981). Rat mammary carcinoma cells secrete active collagenase and activate latent enzyme in the stroma via plasminogen activator. *Int. J. Cancer*, **28**, 509–515.

PAINE, M.L., GIBBINS, J.R., CHEW, K.E., DEMETRIOU, A. & KEFFORD, R.F. (1992). Loss of keratin expression in anaplastic carcinoma cells due to posttranscriptional down-regulation acting in *trans*. *Cancer Res.*, **52**, 6603–6611.

RADINSKY, R., WEISBERG, H.Z., STAROSELSKY, A.N. & FIDLER, I.J. (1992). Expression level of the nm23 gene in clonal populations of metastatic murine and human neoplasms. *Cancer Res.*, **52**, 5808–5814.

RAMSHAW, I.A., CARLSEN, S.A., HOON, D. & WARRINGTON, R.C. (1982). A 6-thioguanine-resistant variant of the 13762 cell line which is no longer tumorigenic or metastatic. *Int. J. Cancer*, **30**, 601–607.

RAMSHAW, I.A. & BADENOCH-JONES, P. (1985). Studies on rat mammary adenocarcinomas: model for metastasis. *Cancer Metastasis Rev.*, **4**, 195–208.

RAMSHAW, I.A., BADENOCH-JONES, P., GRANT, A., MAXTED, M. & CLAUDIANS, C. (1986). Enhanced plasminogen activator production by highly metastatic variant cell lines of a rat mammary adenocarcinoma. *Invasion Metastasis*, **6**, 133-144.

SAMBROOK, J., FRITSCH, E.F. & MANIATIS, T. (1989). *Molecular Cloning: A Laboratory Manual*, Ed. 2. Cold Spring Harbor Laboratory: Cold Spring Harbor, NY.

SASTRE-GARAU, X., LACOMBE, M.L., JOUVE, M., VERON, M. & MAGDELENAT, H. (1992). Nucleoside diphosphate kinase/nm23 expression in breast cancer: lack of correlation with lymph-node metastasis. *In. J. Cancer*, **50**, 533-538.

SHIMADA, N., ISHIKAWA, N., MUNAKATA, Y., TODA, T., WATANABE, K. & KIMURA, N. (1993). A second form (β isoform) of nucleoside diphosphate kinase from rat. *J. Biol. Chem.*, **268**, 2583-2589.

STAHL, J.A., LEONE, A., ROSENGARD, A.M., PORTER, L., KING, C.R. & STEEG. P. (1991). Identification of a second human nm23 gene, nm23-H2. *Cancer Res.*, **51**, 445-449.

STEEG, P.S., BEVILACQUA, G., KOPPER, L., THORGEIRSSON, U.P., TALMADGE, J.E., LIOTTA, L. & SOBEL, M.E. (1988*a*). Evidence for a novel gene associated with low tumor metastatic potential. *J. Natl Cancer Inst.*, **80**, 200-204.

STEEG, P.S., BEVILACQUA, G., POZZATTI, R., LIOTTA, L.A. & SOBEL, M.E. (1988*b*). Altered expression of nm23, a gene associated with low tumor metastatic potential, during adenovirus 2 Ela inhibition of experimental metastasis. *Cancer Res.*, **48**, 6550-6554.

WANG, L., PATEL, U., GHOSH, L., CHEN, H.-C. & BANERJEE, S. (1993). Mutation in the nm23 gene is associated with metastasis in colorectal cancer. *Cancer Res.*, **53**, 717-720.